# A Feasibility Study of Low Cement Content Foamed Concrete Using High Volume of Waste Lime Mud and Fly Ash for Road Embankment

**DOI:** 10.3390/ma15010086

**Published:** 2021-12-23

**Authors:** Zhanchen Li, Huaqiang Yuan, Faliang Gao, Hongzhi Zhang, Zhi Ge, Kai Wang, Renjuan Sun, Yanhua Guan, Yifeng Ling, Nengdong Jiang

**Affiliations:** 1 Shandong Hi-Speed Group Co., Ltd., Jinan 250098, China; sdgslizhanchen@163.com (Z.L.); zzw9000@163.com (K.W.); 2 School of Qilu Transportation, Shandong University, Jinan 250002, China; huaqiangyuan@mail.sdu.edu.cn (H.Y.); 13501054065@139.com (F.G.); hzzhang@sdu.edu.cn (H.Z.); zhige@sdu.edu.cn (Z.G.); sunrenjuan@sdu.edu.cn (R.S.); jiangnengdong@mail.sdu.edu.cn (N.J.); 3 Suzhou Research Institute, Shandong University, Suzhou 215021, China

**Keywords:** foamed concrete, lime mud, fly ash, mechanical properties, hydration heat, calorimetry, microstructure characterization

## Abstract

This paper aims to study the feasibility of low cement content foamed concrete using waste lime mud (LM) and fly ash (FA) as mineral additives. The LM/FA ratio was first optimized based on the compressive strength. Isothermal calorimetry test, ESEM, and XRD were used to investigate the role of LM during hydration. Afterward, the optimized LM/FA ratio (1/5) was used to design foamed concrete with various wet densities (600, 700, 800 and 900 kg/m^3^) and LM–FA dosages (0%, 50%, 60%, 70% and 80%). Flowability measurements and mechanical measurements including compressive strength, flexural strength, splitting strength, elastic modulus, and California bearing ratio were conducted. The results show that the foamed concretes have excellent workability and stability with flowability within 170 and 190 mm. The high alkalinity of LM accelerated the hydration of FA, thereby increasing the early strength. The significant power functions were fitted for the relationships between flexural/splitting and compressive strength with all correlation coefficients (R^2^) larger with 0.95. The mechanical properties of the foamed concrete increased with the density increasing or LM–FA dosage decreasing. The compressive strength, tensile strength, CBR of all prepared foamed concretes were higher than the minimum requirements of 0.8 and 0.15 MPa and 8%, respectively in the standard.

## 1. Introduction

Uneven settlement of embankments significantly reduce the service performance and life of the road. It has been reported that decreasing the additional stress on the subgrade is an efficient solution to control uneven embankment settlement [1,2]. Foamed concrete is a typical lightweight material cellularized by the entrainment of fine air bubbles in the matrix, where the maximum air content can be higher than 80% [3]. It can be designed with a density within the range of 300–1650 kg/m^3^ to have the advantages of self-compacting, lightweight, adjustable compressive strength, and convenient preparation [4,5,6,7]. Accordingly, foamed concrete has been widely used as a new embankment filler to reduce the post-construction settlement and to speed up construction progress [8,9].

The applications of foamed concrete in road engineering have been extensively studied. Huang et al. [10] established a large-scale model of a subgrade filled with foamed concrete to study its long-term performance under cyclic dynamic loads. Test results showed that the foamed concrete with a density within 500–800 kg/m^3^ could meet the requirements of both the static and dynamic conditions. The concrete was still integral after 2 million cyclic loading times, and the cumulative settlement at the top surface of the subgrade was 0.68 mm. She et al. [11] used foamed concrete in the soft foundation of the high-speed railway base in the Hangzhou east railway station project. The settlement, lateral displacement, and soil pressure were continuously tested in the field. Experimental results showed that using foamed concrete could decrease the loading on the soft ground and the settlement of the soft foundation.

However, the most used binding material in foamed concrete is ordinary Portland cement (OPC), which consumes many natural resources and energy, and meanwhile emits roughly 5–7% of the global carbon dioxide, leading to severe environmental and economic problems [12,13,14,15,16]. In recent years, scholars have tried to explore feasible ways to reduce the economic cost and the environmental burden by adding mineral additives [17,18,19,20,21,22]. Fly ash (FA), a byproduct of coal in electric power generating plants, is one of the most commonly used mineral additives. Besides the economic and environmental benefits, FA can improve the workability of the foamed concrete [23]. It has been reported by Wang [24], Chindaprasirt [25], and Amran et al. [26] that the addition of FA reduced drying shrinkage, and improved the transport properties and durability of foamed concrete.

The replacement of cement with FA is generally less than 50%. For the foamed concrete containing high volume FA, a series of problems such as low early strength and easy cracking due to low reactivity of FA have been reported [11,27,28,29]. Therefore, it is necessary to enhance the early reaction in the high volume FA–cement system [30]. Although alkali activators such as caustic soda, aluminate, and aluminosilicate, can be used to improve the early strength containing high volume FA, these activators may increase economic cost and bring other environmental problems.

Lime mud (LM), a byproduct of the alkali recycling process in the paper industry [31] is difficult to be recycled due to the high pH value [32,33]. In general, LM is landfilled or disposed of in the deep sea, which leads to severe soil pollution, groundwater pollution, and marine ecological environment destruction [30]. Due to high alkalinity and carbonate content, LM has the potential to be used as an alkaline activator [34] to accelerate the hydration of mineral additives such as FA and slag, and a filler to dense the microstructure [35]. For instance, Sun et al. [36] found that the hydration process of the alkali-activated slag/FA system was accelerated by the LM. The strength reached 60 MPa at 28 days with 50% slag and FA. However, the existing studies mainly focus on the mechanical properties [37] of normal concrete containing LM–FA. The effect of high volume LM–FA (>50%) on the properties of foamed concrete has not been sufficiently studied.

Therefore, the feasibility of using high volume LM–FA into foamed concrete as a low-cost and sustainable solution for road embankment was studied in this paper. The effect of the FA/LM ratio was first investigated based on the strength of the foamed concrete with 60 wt% LM–FA. Afterward, a total of seventeen sets of foamed concrete mixtures were prepared to study the effect of the mineral additives dosage and density on the workability, compressive strength, flexural strength, splitting strength, elasticity modulus, and California bearing ratio. Besides, the isothermal calorimetry test, ESEM, and XRD were used to investigate the role of LM in hydration. The study provided a guidance for foamed concrete containing high volume of LM–FA in road embankment.

## 2. Materials and Methods

### 2.1. Materials

**Figure 1:**
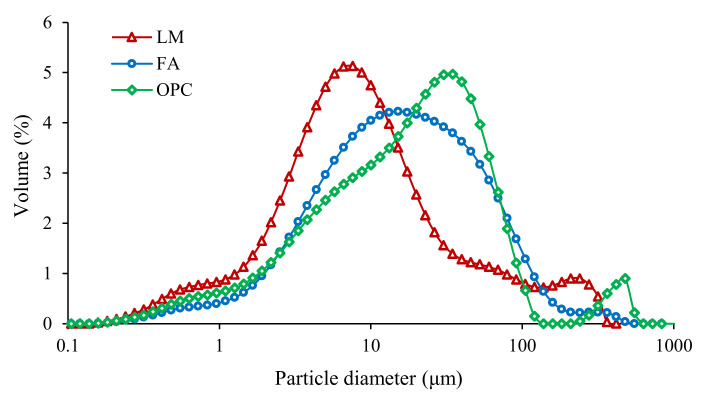
Particle size distribution of LM, FA, and OPC.

The raw materials of foamed concrete include P.O 42.5 cement, FA, LM, and foaming agent. Cement with a density of 3100 kg/m^3^, a specific surface of 358 m^2^/kg, and a standard consistency of 27.9% was used. The specific gravity of FA was 2.4 g/cm^3^. LM was taken from a paper mill (Fangyuan Paper Co., Ltd., Dezhou, China) with a pH value of 13.7. It was dried and ground to an average particle size of 45 μm. The primary chemical compositions of the cementitious materials used are shown in Table 1. Their particle size distributions were measured by a laser particle size analyzer. The results are shown in Figure 1.

**Figure 2:**
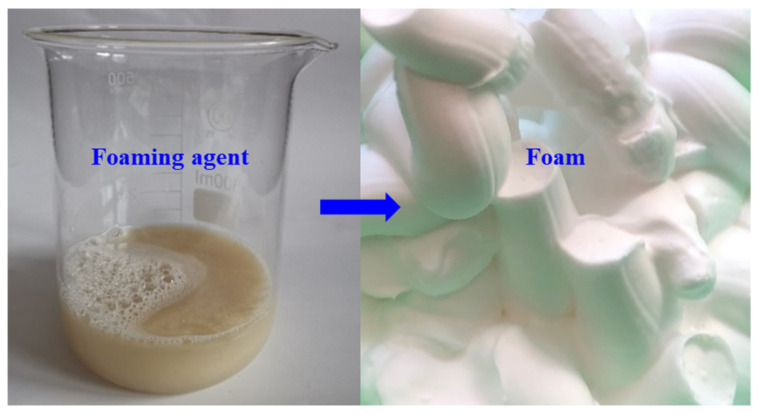
Image of foaming agent and foam.

A protein-based foaming agent with a dilution ratio of 40–50 and a foaming ratio of about 1000–1200 was used. The standard foam density was 40–60 kg/m^3^. Figure 2 shows the foaming agent and foam.

### 2.2. Mixture Proportions of Foamed Concrete

As shown in Table 2, two series of foamed concrete with different LM–FA dosages and target wet densities were designed. Different from normal concrete, the water to binder ratio (w/b) is no longer a major parameter for the strength development of foamed concrete [4,38]. Therefore, a fixed w/b ratio of 0.55 was used to ensure the stability and workability of the mixture. Series I was designed to optimize the ratio of LM/FA. The ratio of LM–FA dosage to binder was 0.6 and the wet density of foamed concrete was 700 kg/m^3^. Polycarboxylate superplasticizer (PS) was utilized to increase the workability of fresh mixtures with a dosage of 0.35% by the mass of LM.

Based on the compressive strength results from Series I, the optimum LM/FA ratio of 1/5 was used to prepare the mixtures in Series II. Foamed concretes with wet densities of 600, 700, 800, and 900 kg/m^3^ were designed. Moreover, the LM–FA dosage of 0%, 50%, 60%, 70%, 80% were used for the mixtures with the wet density of 800 and 900 kg/m^3^. The LM–FA dosages were 0%, 50%, 60% and 70% for the foamed concretes with 700 kg/m^3^ wet density, and 0%, 50%, and 60% for those with 600 kg/m^3^.

### 2.3. Specimen Preparation

A pre-foaming method was used to prepare foamed concrete following the Chinese CJJ/T177−2012 standard [39]. Water was first mixed with the LM for 60 s at a low speed of 145 ± 10 rm. The cement and FA were then added and continuously mixed for 90 s at the low speed and 60 s at a high speed (285 ± 10 rm). Simultaneously, the standard foam with a density of 40 kg/m^3^ was produced through a foam generator in which compressed air and foaming agent were mixed. The mass ratio of the foaming agent to water was 1/49, and the compressed air pressure was 0.4 MPa. Then, the foam was added to the slurry and mixed for 30 s at the low speed. After that, the fresh properties including wet density and flowability were measured before casting. Finally, the mixtures were poured into the mold and covered with a plastic sheet to prevent water evaporation. After 24 h, the samples were stored in a standard curing room (20 °C, 95%RH) until testing.

### 2.4. Testing Methods

#### 2.4.1. Flowability Measurement

The flowability of foamed concrete was measured following the Chinese CJJ/T177-2012 standard [39]. This test measured the spread slump flow without vibration, which is more suitable for construction in the field [40]. In the measurement, a square glass plate (400 mm × 400 mm) and a Φ80 mm× 80 mm cone were used. The cone was filled with the fresh mixture and lifted vertically within 3 s. The average spread diameter at four equally divided directions was reported for each mixture.

#### 2.4.2. Density Measurement

Wet density was calculated according to the fresh foamed concrete weight and the given volume of the standard vessels. The dry density of the specimen (100 mm × 100 mm × 100 mm) was measured after curing under standard conditions for 28 days. Before testing, each specimen was dried in an oven at 60 °C till constant weight.

#### 2.4.3. Mechanical Properties Measurement

Compressive strength, flexural strength, tensile strength, and modulus of elasticity were measured following the Chinese GB/T 11969-2008 standard [41]. California bearing ratio (CBR) measurement was performed following the Chinese JTG E40-2007 standard [42]. In this test, a standard piston with a diameter of 50 mm was used to penetrate the specimen at a standard rate of 1 mm/min. CBR is the ratio between the test load applied on the specimen and the standard load at the penetration of 2.5 mm, expressed as a percentage. The reported result was the average of three specimens for all properties. Table 3 lists the basic parameters of these mechanical tests.

#### 2.4.4. Isothermal Calorimetry Test

An eight-channel isothermal calorimeter model I-Cal 8000HPC was used to measure the heat evolution. Seven types of binder mixtures were prepared for the test. The LM–FA dosages of 0%, 60%, and 70% were used with the LM/FA ratios of 0/1, 1/5, and 1/1. A water-to-binder ratio of 0.55 was used for all mixtures. After mixing, the fresh mixture was cast into a glass thermos and then placed in the calorimeter chamber. The heat evolution rate was recorded continually up to 168 h (7 days) for the analysis.

#### 2.4.5. ESEM and XRD

The pore structure and morphology of hydration products of foamed concretes were observed by environmental scanning electron microscopy (ESEM). Specimen of 10 × 10 × 5 mm from the center of foamed concrete was polished and dried. In total, 25.0 kV accelerating voltage was used during the scan, and 30×, 500×, and 2500× ESEM images were taken using an ESEM.

X-ray diffractometer (XRD) was used to measure mineral composition phases of the foamed concrete with the LM–FA dosages of 0% and 70% at 28 days. Cu-Ka radiation at 40 kV and 30 mA with a scanning speed of 0.02°/s over a range of 10–90° were used during the measurement.

## 3. Results

### 3.1. Fresh Properties of Foamed Concrete

Table 4 shows the measured fresh properties of foamed concrete. A density ratio is defined as the measured wet density/targeted wet density. This ratio was close to one for all mixtures, which indicated that no severe foam crush or segregation occurred during the preparation of foamed concrete. The flowability was between 170 and 190 mm for all mixtures, which met the specification in the Chinese JTG D30−2015 standard for the in-situ foamed lightweight subgrade [43].

In addition, the mixtures in Series Ⅰ had relatively consistent densities, indicating that the ratio of LM/FA had a negligible effect on the stability of the foamed concrete. In Series II, as expected, the dry density was positively correlated with the wet density. However, for the mixtures with the same wet density, the higher LM–FA dosage came the lower dry density due to less requirement of water for FA hydration. Specifically, the bound water in hydration products decreased, and more free water remained in the pores of the specimen. Therefore, the free water evaporated during the drying process, leading to a lower dry density.

### 3.2. Influence of LM/FA Ratio on the Compressive Strength of Foamed Concrete

**Figure 3:**
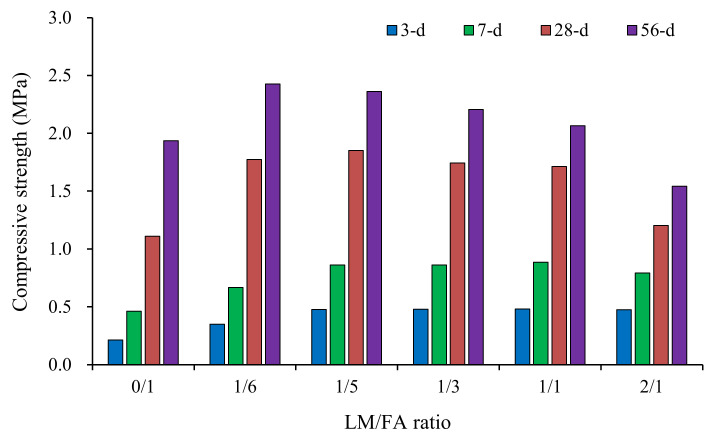
Compressive strengths of Series I mixtures.

The compressive strength results of foamed concrete in Series I at different curing ages are shown in Figure 3. It shows that the LM/FA ratio had a significant effect on compressive strength. The early compressive strength increased with LM/FA ratio increasing to 1/5 and stabilizing afterward. This is due to that the high pH of LM enhanced the activity of FA, thus accelerating the hydration process and improving the early strength. The 28-day compressive strengths first increased and then decreased with LM/FA ratio increasing. Compared with the mixture without LM, the compressive strength of the mixture with 1/5 LM/FA ratio increased the most. Therefore, the optimal ratio of LM/FA was determined as 1/5 and further used for the foamed concretes in Series II.

### 3.3. Compressive Strengths of Foamed Concrete

The compressive strength results of the foamed concretes in Series Ⅱ are shown in Table 5. The compressive strength was significantly dependent on the wet density and LM–FA dosage. An increased LM–FA dosage gradually decreased the compressive strength of foamed concrete. For the foamed concretes of 700 kg/m^3^ wet density, as the LM–FA dosage increased from 0% to 70%, the 28-day compressive strength decreased by 1.1 MPa. However, the 28-day compressive strength of all mixtures was higher than 0.8 MPa which met the technical specification (JTG D30−2015) [43] for highway embankment design.

**Figure 4:**
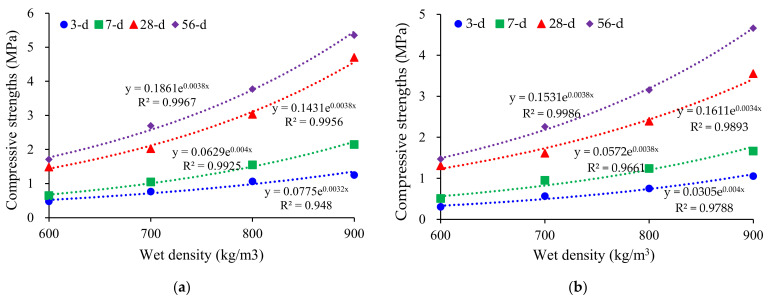
Relationship between the wet density and strength of the mixture: (**a**) LM–FA dosage of 50%; (**b**) LM–FA dosage of 60%.

In addition, density had a significant effect on compressive strength, i.e., a higher density considerably increased compressive strength. It can be attributed to the following two reasons: (1) The foamed concrete with a high wet density had a minor porosity and a thicker pore wall; (2) The internal pore structure was more integrated, resulting in the densification of the cell wall paste structure. Figure 4 shows the relationship between the compressive strength and wet density of the mixtures with LM–FA dosages of 50% and 60%. It was expressed by an exponential equation [26,44], with a correlation coefficient (*R*^2^) higher than 0.95. Similar trends have been reported in previous research [4,45].

### 3.4. Tensile Strength of Foamed Concrete

**Figure 5:**
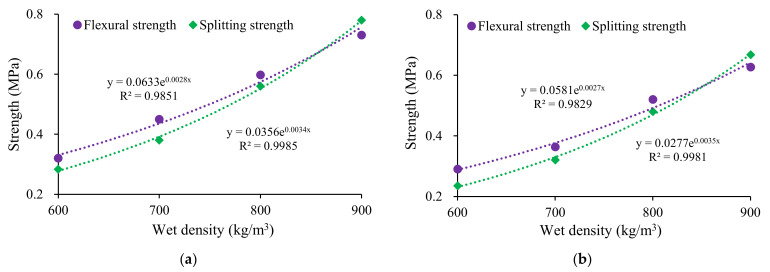
Influence of wet density on tensile properties of mixtures: (**a**) LM–FA dosage of 50%; (**b**) LM–FA dosage 60%.

Table 6 summarizes the 28-day flexural strength and splitting strength of Series II mixtures. Clearly, the flexural strength and splitting strength decreased with the LM–FA dosage increasing. Comparing 70% LM–FA dosage with 0% LM–FA, the flexural strength and splitting strength of foamed concrete with a density of 700 kg/m^3^ decreased by 0.21 MPa and 0.18 MPa, respectively. As shown in Figure 5, the splitting and flexural strength significantly increased with the wet density increasing. An exponential equation could describe the relationship between compressive strength and wet density.

Although the tensile strength (flexural strength and splitting strength) of foamed concrete is much lower than that of ordinary concrete, this does not apply to the ratio of tensile/compressive strength [7]. The ratio of flexural/compressive strength of the foamed concrete was between 0.15 and 0.23, and the ratio of splitting/compressive strength was between 0.16 and 0.21. Both ratios were higher than those of ordinary concrete (between 0.08 and 0.11 [40]). Notably, the tensile strength of the mixtures in Series Ⅱ was higher than 0.15 MPa, which satisfies the requirements of the roadbed design standard [42].

**Figure 6:**
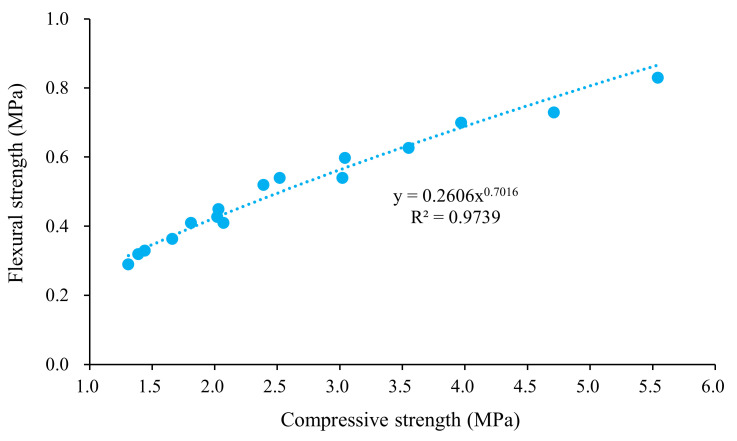
Relationship between flexural and compressive strength of the mixture.

**Figure 7:**
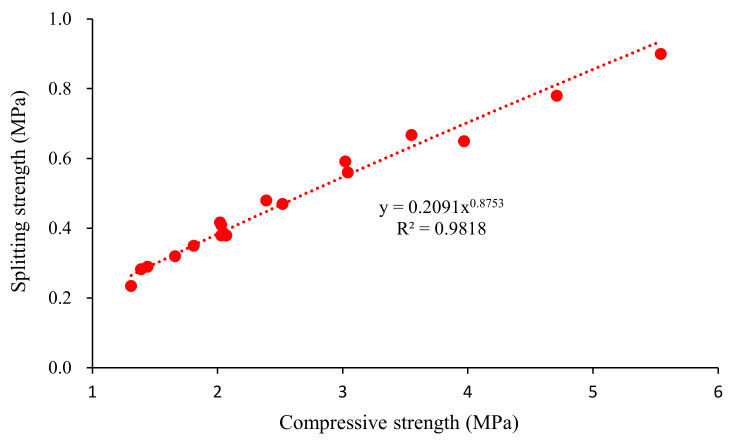
Relationship between splitting and compressive strength of the mixture.

Figure 6 shows the relationship between flexural strength and compressive strength at 28 days. The relationship between the splitting strength and the compressive strength is shown in Figure 7. The results show that the flexural strength was significantly increased with the compressive strength increasing. There was a significant power function relationship between them. The determination coefficients (R^2^) of the fits were all above 0.97. As shown in Table 7, the formulas were used to estimate the splitting strength of concrete based on the compressive strength. However, the performance of foamed concrete was significantly affected by the binder type and pore structure. Thus, the fitting parameters were different from those in the standards.

### 3.5. Elastic Modulus

**Figure 8:**
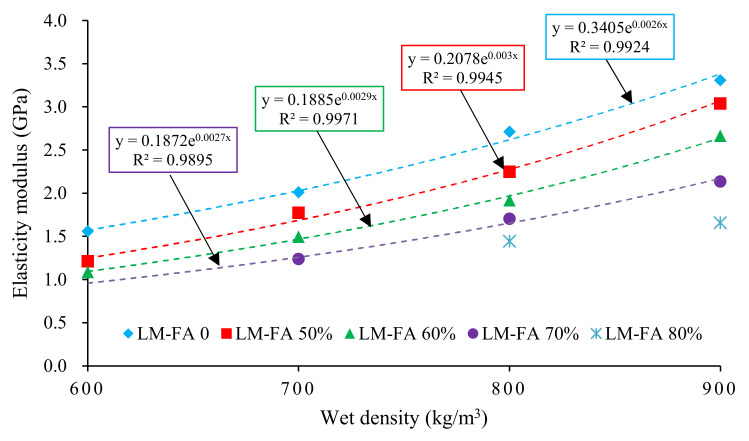
The elasticity modulus of Series Ⅱ mixtures.

Figure 8 shows the elastic modulus of mixtures in Series II. It reveals that the elastic modulus of the foamed concrete was between 1.1 and 3.3 GPa. The wet density greatly affected the elastic modulus. There is a significant power function relationship between the elastic modulus and the wet density. Foamed concrete with 80% LM–FA (MA80) was not fitted due to the small amount of test data. This is because the internal pores were smaller for the mixtures with higher densities, and their spacing became relatively larger, thus leading to a robust interlocking system and an increased load transfer capability [36].

**Figure 9:**
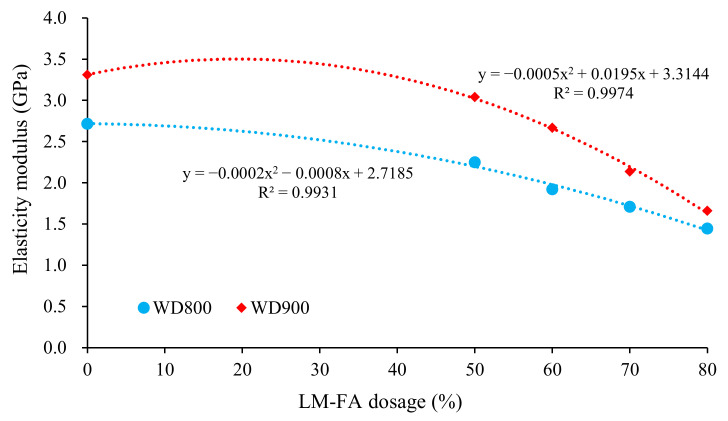
Influence of LM–FA dosage on the elastic modulus of mixtures.

Additionally, the elastic modulus decreased gradually with the LM–FA dosage increasing. Foamed concrete with a wet density of 600 kg/m^3^ and 60% LM–FA (WD600MA60) has the lowest elastic modulus of about 1.1 GPa. The measured results show that all the mixtures in Series II met the requirement of technical specification (JTG D30−2015) [43] for elastic deformation capacity. Besides, Figure 9 indicates that the elastic modulus was in a significant quadratic function of LM–FA dosage. The goodness of fit (R^2^) was above 0.99. Moreover, the elastic modulus of the foamed concrete with a higher density was more sensitive to LM–FA dosage.

**Figure 10:**
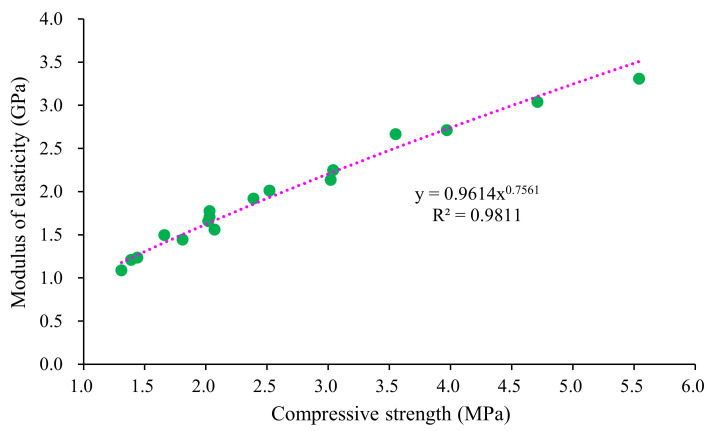
Relationship between elasticity modulus and compressive strength of the mixture.

Figure 10 presents the relationship between the elastic modulus and 28-day compressive strength. As seen in Figure 10, a power function was used to describe such a relationship with R^2^ greater than 0.95 [5,26].

### 3.6. California Bearing Ratio

California load ratio is a measurement of the resistance of road materials to local load. It is commonly used to evaluate the bearing capacity of subgrade materials. Table 8 shows the influence of the LM–FA dosage on CBR of foamed concrete. It can be seen that the increased LM–FA dosage reduced CBR. For the foamed concrete with 700 kg/m^3^ wet density, compared to the specimen without LM–FA, the CBR decreased by 38.5%, 54.3%, and 68.0% for the 50%, 60%, and 70% LM–FA dosage, respectively. Foamed concrete with a wet density of 600 kg/m^3^ and 60% LM–FA (WD600MA60) had the smallest CBR of 13.5%. It met the requirement of the Chinese JTG D30-2015 [43] standard of CBR (>8%) for the embankment filler.

**Figure 11:**
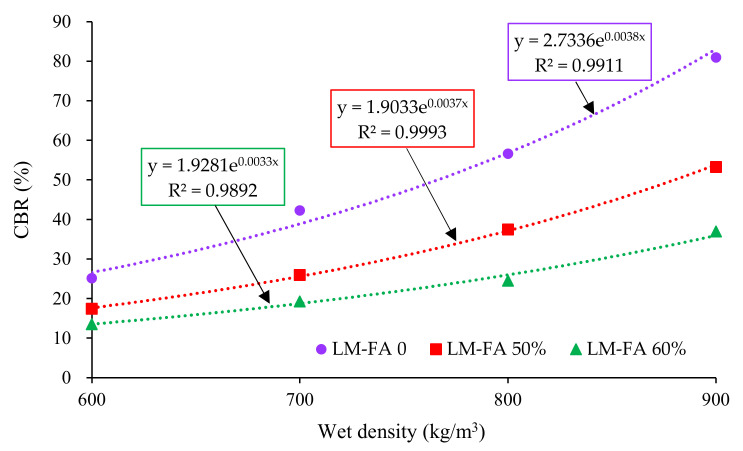
Relationship between the wet density and CBR of mixtures.

Figure 11 shows the effect of wet density on CBR of foamed concrete. Regardless of LM–FA dosage, CBR increased rapidly with wet density increasing. A significant exponential relationship was found between the CBR and wet density because the porosity and pore structure played a significant role in the mechanical properties of foamed concrete. The exponent in the function increased with the LM–FA dosage decreasing, indicating that CBR of the mixtures with lower LM–FA dosages grew faster. These empirical equations can estimate CBR of foamed concrete with different wet densities in practical application.

### 3.7. Calorimetry and Microstructure Characterization

**Figure 12:**
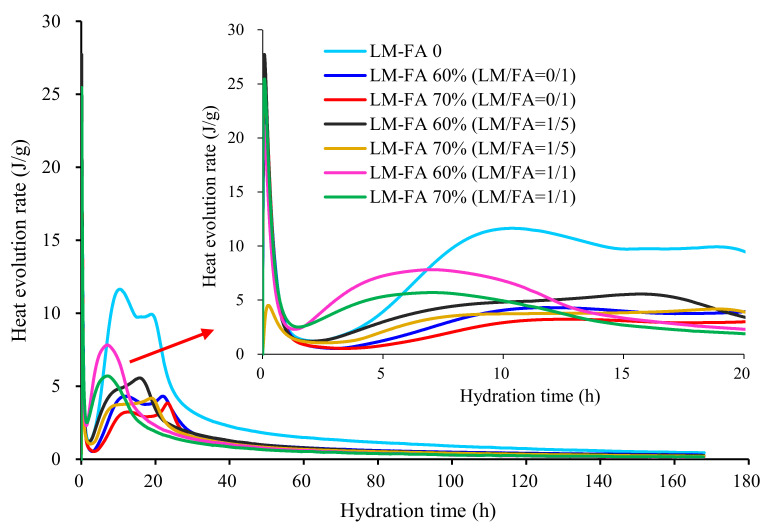
Influence of LM and FA on thermal power of mixtures.

**Figure 13:**
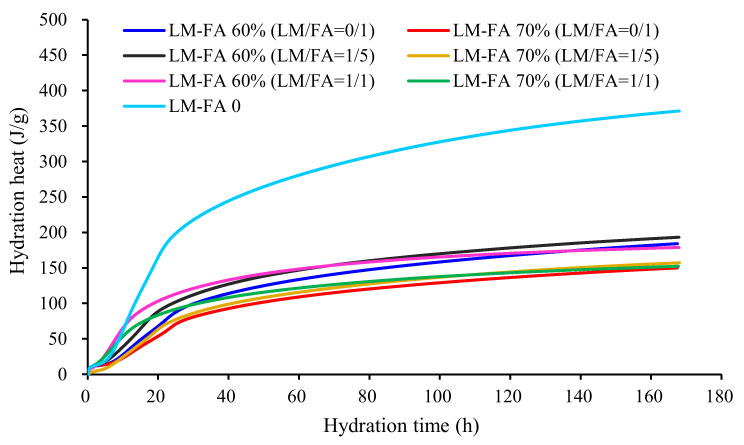
Influence of LM and FA on hydration heat of mixtures.

Figure 12 shows the hydration heat release rate of foamed concrete with different LM–FA dosages. The patterns of hydration process for all mixtures were similar. According to the characteristics of hydration heat release, five phases were distinguished: the pre-induction phase, induction phase, acceleration phase, deceleration phase, and stable phase. Obviously, replacing cement with high volume FA, the peak of the hydration heat release rate was significantly reduced and postponed in the acceleration period. It confirms that the high volume of FA significantly reduced the hydration heat but delayed the early hydration, leading to lower early-age mechanical properties. When LM was used (LM/FA = 1/5, LM–FA dosages = 60%, 70%), the induction phase and acceleration phase moved forward, and the peak of these phases increased. As shown in Figure 13, the cumulative heat of cement–FA binder increased by adding LM. It is because the high alkalinity of LM corroded the surface of FA, thus exposing SiO_2_ and accelerating the hydration process [29]. 

On the other hand, for a given dosage of LM–FA, the cumulative heat of binder in the early hydration stage increased with LM/FA ratio increasing. However, as the hydration time increased, the binder with 1/5 LM/FA ratio possessed a lower accumulated heat of hydration. It explains that the long-term strength decreased of foamed concrete with a higher LM/FA ratio.

**Figure 14:**
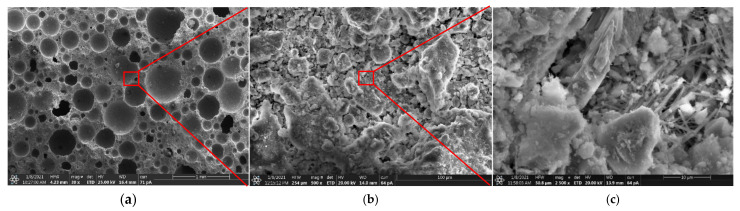
SE images of pore structure and hydration products foamed concrete: (**a**) magnification of 30×; (**b**) magnification of 500×; (**c**) magnification of 2500×.

Figure 14 shows the pore structure and hydration products of foamed concrete. It is clear that most pores were complete sphere shape, and a negligible number of pores were connected, indicating that no severe bleeding and segregation happened during the preparation. Most pore diameters were less than 0.5 mm, and only a few pores are larger than 0.5 mm. Despite LM accelerated the hydration of FA, a significant amount of unreacted FA particles was still observed in the specimens cured for 28 days. However, there were some hydration products of cement attached to the surface of FA particles. Besides, it can be seen that LM particles filled in the interstitial space of hydration products of OPC and FA, which was favorable to form a dense microstructure and improve the strength.

**Figure 15:**
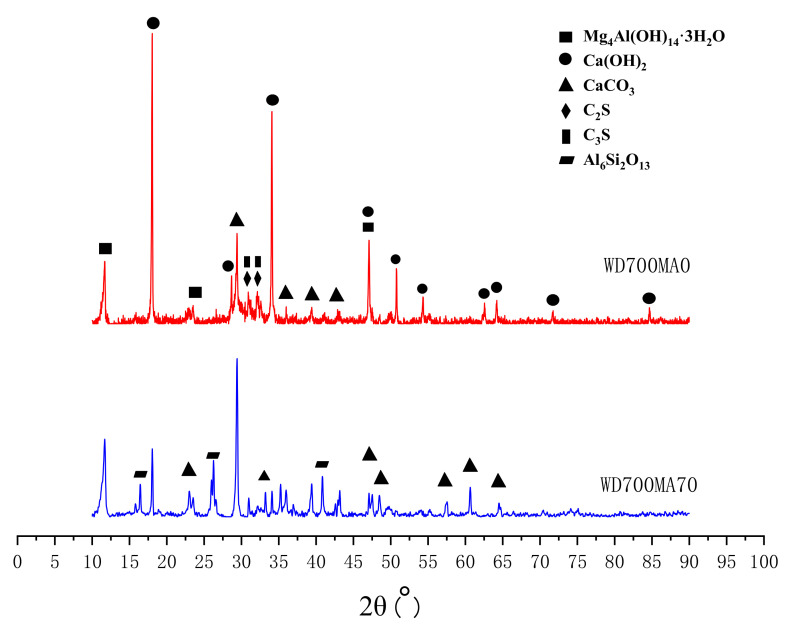
XRD patterns of foamed concrete with different LM–FA dosages.

The XRD patterns of foamed concrete with different LM–FA dosages are shown in Figure 15. The main crystalline phase was portlandite (Ca(OH)_2_) after 28-day curing. Besides, calcium silicate hydrates (C-S-H), calcite (CaCO_3_), unreacted dicalcium silicate (C_2_S), and tricalcium silicate (C_3_S) were also found in the samples. Replaced cement with 70% LM–FA, the diffraction intensity of calcite significantly increased, and that of portlandite was reduced. Mullite, one of the mineral phases of FA, was also found. The hydration of FA consumed a large amount of Ca(OH)_2_ from cement hydration products and LM, thus resulting in an enhancement on the pozzolanic reaction of FA.

## 4. Conclusions

In this paper, the feasibility of utilizing high volume of LM–FA foamed concrete as a lightweight filling material for embankment was investigated. The mixtures with various wet densities and LM–FA dosages were prepared and tested. Based on the experimental analysis, the following conclusions can be drawn:

(1) The high alkalinity of LM stimulated the activity of FA, thereby enhancing the mechanical properties of the foamed concretes with high LM–FA dosages at an early age. The contribution of LM to the long-term strength is mainly due to its filling effect. The optimal LM/FA ratio was 1/5.
(2) An exponential equation could describe the relationship between compressive strength and wet density. Both the splitting strength and flexural strength have a significant power function relationship with the compressive strength. The determination coefficients (R^2^) of all equations were above 0.95.
(3) Both the elasticity modulus and CBR grow exponentially with the wet density increasing. A higher LM–FA dosage led to a larger increased rate of elasticity modulus and CBR.
(4) The mechanical properties of foamed concrete increased with the wet density increasing or LM–FA dosage decreasing. The compressive strength, tensile strength, CBR of all mixtures were higher than the minimum requirement of 0.8 MPa, 0.15 MPa, and 8%, respectively.

The effects of LM–FA dosage and wet density on the shrinkage, durability, and microstructure of foamed concrete will be studied in the future.

## Figures and Tables

**Table 1:** Chemical compositions of the raw materials.

Materials	SiO_2_	Al_2_O_3_	Fe_2_O_3_	CaO	MgO	SO_3_	Na_2_O	K_2_O	LOI
OPC	21.96	4.73	3.68	64.63	2.59	0.3	0.43	0.2	2.13
FA	48.54	34.68	5.23	2.6	0.459	1.12	−	−	4.67
LM	2.08	0.71	0.36	47.5	2.74	−	2.8	0.27	42.84

**Table 2:** Mixture proportions of foamed concrete.

Series	Mixture Code	w/b	Targeted Wet Density (kg/m^3^)	LM–FA Dosage (%)	LM/FA	OPC (kg/m^3^)	FA (kg/m^3^)	LM (kg/m^3^)	Water (kg/m^3^)	PS (kg/m^3^)	Foam (kg/m^3^)
I	LM0	0.55	700	60	0/1	172	258	0	237	0	32.4
	LM14	0.55	700	60	1/6	172	221	37	237	0.13	32.4
	LM17	0.55	700	60	1/5	172	215	43	237	0.15	32.4
	LM25	0.55	700	60	1/3	172	194	65	237	0.23	32.4
	LM50	0.55	700	60	1/1	172	129	129	237	0.45	32.4
	LM67	0.55	700	60	2/1	172	86	172	237	0.60	32.4
II	WD600MA0	0.55	600	0	—	363	0	0	200	0	38.2
	WD600MA50	0.55	600	50	1/5	182	152	30	200	0.11	36.9
	WD600MA60	0.55	600	60	1/5	146	182	36	200	0.13	36.6
	WD700MA0	0.55	700	0	—	429	0	0	236	0.00	34.4
	WD700MA50	0.55	700	50	1/5	215	179	36	237	0.13	32.8
	WD700MA60	0.55	700	60	1/5	172	215	43	237	0.15	32.4
	WD700MA70	0.55	700	70	1/5	129	251	50	237	0.18	32.2
	WD800MA0	0.55	800	0	—	496	0	0	273	0	31.3
	WD800MA50	0.55	800	50	1/5	249	207	41	273	0.14	29.6
	WD800MA60	0.55	800	60	1/5	199	249	50	274	0.18	29.4
	WD800MA70	0.55	800	70	1/5	149	290	58	274	0.20	29.2
	WD800MA80	0.55	800	80	1/5	100	332	66	274	0.23	28.7
	WD900MA0	0.55	900	0	—	563	0	0	309	0	28.3
	WD900MA50	0.55	900	50	1/5	282	235	47	310	0.16	26.9
	WD900MA60	0.55	900	60	1/5	226	282	56	310	0.20	26.3
	WD900MA70	0.55	900	70	1/5	169	329	66	310	0.23	25.8
	WD900MA80	0.55	900	80	1/5	113	376	75	311	0.26	25.1

Note: WD represents the wet density; MA represents mineral admixture (lime mud and fly ash) dosage.

**Table 3:** Parameters for the performance test of the mixture.

Properties	Specimens Dimension	Curing Time (d)	Loading Speed
Compressive strength	100 mm × 100 mm × 100 mm	3, 7, 28, 56	2.0 kN ± 0.5 kN/s
Flexure strength	100 mm × 100 mm × 400 mm	28	0.2 kN ± 0.05 kN/s
Splitting strength	100 mm × 100 mm × 100 mm	28	0.2 kN ± 0.05 kN/s
Elastic modulus	100 mm × 100 mm × 300 mm	28	−
CBR	Φ 150 mm× 170 mm	7	1 mm/min

**Table 4:** Measured fresh properties and dry density of the mixtures.

Series	Mixture Code	Targeted Wet Density (kg/m^3^)	Measured Wet Density (kg/m³)	Density Ratio	Flowability (mm)	Dry Density (kg/m^3^)
I	LM0	700	702	1.003	185	503
	LM14	700	701	1.001	181	502
	LM17	700	697	0.996	179	499
	LM25	700	695	0.993	185	498
	LM50	700	699	0.999	183	501
	LM67	700	703	1.004	175	504
II	WD600MA0	600	594	0.990	173	453
	WD600MA50	600	604	1.007	181	422
	WD600MA60	600	598	0.997	179	411
	WD700MA0	700	695	0.993	181	545
	WD700MA50	700	702	1.002	188	518
	WD700MA60	700	702	1.003	185	503
	WD700MA70	700	706	1.009	184	482
	WD800MA0	800	800	1.000	181	643
	WD800MA50	800	808	1.010	190	590
	WD800MA60	800	798	0.998	180	576
	WD800MA70	800	802	1.003	185	560
	WD800MA80	800	800	1.000	183	545
	WD900MA0	900	891	0.990	188	745
	WD900MA50	900	908	1.009	172	680
	WD900MA60	900	900	1.000	181	661
	WD900MA70	900	900	1.000	185	643
	WD900MA80	900	896	0.996	185	612

**Table 5:** Compressive strength of Series Ⅱ mixtures.

Mixtures	Wet Density (kg/m^3^)	LM–FA Dosage (%)	Compressive Strengths			
			3 d	7 d	28 d	56 d
WD600MA0	600	0	0.99	1.30	2.07	2.54
WD600MA50	600	50	0.48	0.65	1.39	1.71
WD600MA60	600	60	0.31	0.51	1.31	1.47
WD700MA0	700	0	1.50	1.96	2.52	3.06
WD700MA50	700	50	0.77	1.05	2.03	2.70
WD700MA60	700	60	0.56	0.95	1.66	2.26
WD700MA70	700	70	0.42	0.66	1.44	1.65
WD800MA0	800	0	2.54	3.21	3.97	4.78
WD800MA50	800	50	1.07	1.56	3.04	3.78
WD800MA60	800	60	0.75	1.24	2.39	3.15
WD800MA70	800	70	0.48	1.10	2.03	2.73
WD800MA80	800	80	0.31	0.71	1.81	1.98
WD900MA0	900	0	3.28	4.29	5.54	6.22
WD900MA50	900	50	1.26	2.15	4.71	5.36
WD900MA60	900	60	1.05	1.67	3.55	4.66
WD900MA70	900	70	0.73	1.39	3.02	4.05
WD900MA80	900	80	0.37	0.87	2.02	3.01

**Table 6:** Flexural and splitting strength of Series II mixtures.

Mixtures	Flexural Strength (MPa)	Splitting Strength (MPa)	Flexural Strength/Compressive Strength	Splitting Strength/Compressive Strength
WD600MA0	0.41	0.38	0.198	0.184
WD600MA50	0.32	0.28	0.230	0.204
WD600MA60	0.29	0.25	0.221	0.191
WD700MA0	0.54	0.47	0.214	0.187
WD700MA50	0.45	0.38	0.222	0.187
WD700MA60	0.36	0.32	0.219	0.193
WD700MA70	0.33	0.29	0.229	0.201
WD800MA0	0.70	0.65	0.176	0.164
WD800MA50	0.60	0.56	0.197	0.184
WD800MA60	0.52	0.48	0.218	0.201
WD800MA70	0.45	0.41	0.222	0.202
WD800MA80	0.41	0.35	0.227	0.193
WD900MA0	0.83	0.90	0.150	0.162
WD900MA50	0.73	0.78	0.155	0.166
WD900MA60	0.63	0.67	0.177	0.188
WD900MA70	0.54	0.59	0.179	0.196
WD900MA80	0.43	0.42	0.212	0.207

**Table 7:** Formulas are suggested by the model code.

Code	Formula
American Concrete Institute	*f*_t,tp_ = 0.59(*f*_c_)^0.5^
CEB-FIP Model Code: Design Code	*f*_t,tp_ = 0.301(*f*_c_)^0.67^

Note: *f*_t,tp_ is the splitting tensile strength of the specimen, MPa; *f*_c_ is the compressive strength of the specimen, MPa.

**Table 8:** Influence of LM–FA dosage on the CBR of mixtures.

Mixture	LM–FA Dosage (%)				
	0	50	60	70	80
WD600	25.21	17.46	13.56	-	-
WD700	42.31	26.00	19.32	13.72	-
WD800	56.67	37.49	24.56	18.56	13.95
WD900	81.00	53.31	37.00	30.53	18.48

## Data Availability

The data presented in this study are available on request from the corresponding author.

## Abbreviations

FA	Fly ash
LM	Lime mud
WD	Wet density
MA	Mineral additives
OPC	Ordinary Portland cement
PS	Polycarboxylate superplasticizer
CBR	California bearing ratio
XRD	X-ray diffractometer
ESEM	Environmental scanning electron microscopy

## References

[1] Kim T.H., Kang G.C. (2013). Performance evaluation of road embankment constructed using lightweight soils on an unimproved soft soil layer. Eng. Geol..

[2] Shi X., Huang J., Su Q. (2020). Experimental and numerical analyses of lightweight foamed concrete as filler for widening embankment. Constr. Build. Mater..

[3] Song Y., Lange D. (2021). Influence of fine inclusions on the morphology and mechanical performance of lightweight foam concrete. Cem. Concr. Compos..

[4] Ramamurthy K., Kunhanandan Nambiar E.K., Indu Siva Ranjani G. (2009). A classification of studies on properties of foam concrete. Cem. Concr. Compos..

[5] Pan Z., Li H., Liu W. (2014). Preparation and characterization of super low density foamed concrete from Portland cement and admixtures. Constr. Build. Mater..

[6] Raj A., Sathyan D., Mini K.M. (2019). Physical and functional characteristics of foam concrete: A review. Constr. Build. Mater..

[7] Zhang H., Qi X., Ma C., Wu J., Bi Y., Sun R., Yu J., Xie D., Song J. (2020). Effect Analysis of Soil Type and Silt Content on Silt-Based Foamed Concrete with Different Density. Materials.

[8] Wu J., Lv C., Pi R., Zhang H., Bi Y., Song X., Wang Z. (2021). The stability and durability of silt-based foamed concrete: A new type of road engineering material. Constr. Build. Mater..

[9] Decký M., Drusa M., Zgútová K., Blaško M., Hájek M., Scherfel W. (2016). Foam Concrete as New Material in Road Constructions. Procedia Eng..

[10] Huang J.J., Su Q., Zhao W.H., Li T., Zhang X.X. (2017). Experimental study on use of lightweight foam concrete as subgrade bed filler of ballastless track. Constr. Build. Mater..

[11] She W., Du Y., Zhao G., Feng P., Zhang Y., Cao X. (2018). Influence of coarse fly ash on the performance of foam concrete and its application in high-speed railway roadbeds. Constr. Build. Mater..

[12] Zhang H., Xu Y., Gan Y., Chang Z., Schlangen E., Šavija B. (2020). Microstructure informed micromechanical modelling of hydrated cement paste: Techniques and challenges. Constr. Build. Mater..

[13] Zhang H., Xu Y., Gan Y., Chang Z., Schlangen E., Šavija B. (2019). Combined experimental and numerical study of uniaxial compression failure of hardened cement paste at micrometre length scale. Cem. Concr. Res..

[14] Wang D., Wang Q., Huang Z. (2020). New insights into the early reaction of NaOH-activated slag in the presence of CaSO4. Compos. B Eng..

[15] Zhang J., Chen T., Gao X. (2021). Incorporation of self-ignited coal gangue in steam cured precast concrete. J. Clean. Prod..

[16] Bilal H., Chen T., Ren M., Gao X., Su A. (2021). Influence of silica fume, metakaolin & SBR latex on strength and durability performance of pervious concrete. Constr. Build. Mater..

[17] Farzampour A. (2019). Compressive behavior of concrete under environmental effects. Compressive Strength of Concrete.

[18] Chalangaran N., Farzampour A., Paslar N., Fatemi H. (2021). Experimental investigation of sound transmission loss in concrete containing recycled rubber crumbs. Adv. Concr. Constr..

[19] Navid C., Alireza F., Nima P. (2020). Nano Silica and Metakaolin Effects on the Behavior of Concrete Containing Rubber Crumbs. CivilEng.

[20] Petrounias P., Rogkala A., Giannakopoulou P.P., Lampropoulou P., Koutsovitis P., Koukouzas N., Laskaris N., Pomonis P., Hatzipanagiotou K. (2020). Removal of Cu (II) from industrial wastewater using mechanically activated serpentinite. Energies.

[21] Petrounias P., Giannakopoulou P.P., Rogkala A., Kalpogiannaki M., Koutsovitis P., Damoulianou M.-E., Koukouzas N. (2020). Petrographic characteristics of sandstones as a basis to evaluate their suitability in construction and energy storage applications. A case study from Klepa Nafpaktias (Central Western Greece). Energies.

[22] Zhuang S., Wang Q. (2021). Inhibition mechanisms of steel slag on the early-age hydration of cement. Cem. Concr. Res..

[23] Kunhanandan Nambiar E.K., Ramamurthy K. (2006). Influence of filler type on the properties of foam concrete. Cem. Concr. Compos..

[24] Wang L., Yang H.Q., Zhou S.H., Chen E., Tang S.W. (2018). Mechanical properties, long-term hydration heat, shinkage behavior and crack resistance of dam concrete designed with low heat Portland (LHP) cement and fly ash. Constr. Build. Mater..

[25] Chindaprasirt P., Jaturapitakkul C., Sinsiri T. (2005). Effect of fly ash fineness on compressive strength and pore size of blended cement paste. Cem. Concr. Compos..

[26] Amran Y.H.M., Farzadnia N., Abang Ali A.A. (2015). Properties and applications of foamed concrete; a review. Constr. Build. Mater..

[27] Jitchaiyaphum K., Sinsiri T., Jaturapitakkul C., Chindaprasirt P. (2013). Cellular lightweight concrete containing high-calcium fly ash and natural zeolite. Int. J. Miner. Met. Mater..

[28] Kearsley E.P., Wainwrightb P.J. (2001). The effect of high fly ash content on the compressive strength of foamed concrete. Cem. Concr. Res..

[29] Kipkemboi B., Zhao T., Miyazawa S., Sakai E., Nito N., Hirao H. (2020). Effect of C3S content of clinker on properties of fly ash cement concrete. Constr. Build. Mater..

[30] Park S.S., Kang H.Y. (2006). Strength and microscopic characteristics of alkali-activated fly ash-cement. Korean J. Chem. Eng..

[31] Sun R., Li Y., Liu C., Xie X., Lu C. (2013). Utilization of lime mud from paper mill as CO_2_ sorbent in calcium looping process. Chem. Eng. J..

[32] He X., Xu W., Sun W., Ni J. (2013). Phosphate removal using compounds prepared from paper sludge and fly ash. Environ. Earth Sci..

[33] Ye C., Yan B., Ji X., Liao B., Gong R., Pei X., Liu G. (2019). Adsorption of fluoride from aqueous solution by fly ash cenospheres modified with paper mill lime mud: Experimental and modeling. Ecotoxicol. Environ. Saf..

[34] Jena S.K., Dash N., Rath S.S. (2019). Effective utilization of lime mud for the recovery of potash from mica scraps. J. Clean. Prod..

[35] Cwirzen A., Provis J.L., Penttala V., Habermehl-Cwirzen K. (2014). The effect of limestone on sodium hydroxide-activated metakaolin-based geopolymers. Constr. Build. Mater..

[36] Sun R., Fang C., Zhang H., Ling Y., Feng J., Qi H., Ge Z. (2021). Chemo-mechanical properties of alkali-activated slag/fly ash paste incorporating white mud. Constr. Build. Mater..

[37] Huang H., Gao X., Teng L. (2021). Fiber alignment and its effect on mechanical properties of UHPC: An overview. Constr. Build. Mater..

[38] Ge Z., Feng Y., Yuan H., Zhang H., Sun R., Wang Z. (2021). Durability and shrinkage performance of self-compacting concrete containing recycled fine clay brick aggregate. Constr. Build. Mater..

[39] CJJ/T 177-2012 (2012). Technical Specification for Foamed Mixture Lightweight Soil Filling Engineering.

[40] Ge Z., Yuan H., Sun R., Zhang H., Wang W., Qi H. (2020). Use of green calcium sulphoaluminate cement to prepare foamed concrete for road embankment: A feasibility study. Constr. Build. Mater..

[41] GB/T 11969-2008 (2008). Test Methods of Autoclaved Aerated Concrete.

[42] JTG E40—2007 (2007). Test Methods of Soils for Highway Engineering.

[43] JTG D30 (2015). Specification for Design of Highway Subgrades.

[44] Just A., Middendorf B. (2009). Microstructure of high-strength foam concrete. Mater. Charact..

[45] Kunhanandan Nambiar E.K., Ramamurthy K. (2007). Models for strength prediction of foam concrete. Mater. Struct..

